# Age-related changes in serum reproductive hormone levels and prevalence of androgen deficiency in Chinese community-dwelling middle-aged and aging men: Two cross-sectional studies in the same population

**DOI:** 10.1097/MD.0000000000018605

**Published:** 2020-01-03

**Authors:** Shan-Jie Zhou, Ming-Jia Zhao, Yi-Hong Yang, Di Guan, Zhi-Guang Li, Yu-Dang Ji, Bao-Long Zhang, Xue-Jun Shang, Cheng-Liang Xiong, Yi-Qun Gu

**Affiliations:** a Reproductive Medicine Center, Department of Gynecology and Obstetrics, Peking University International Hospital, Beijing; b Department of Reproduction and Genetics, Maternity and Child Health Care Hospital of Tangshan, Tangshan; c Reproductive Medicine Centre, Department of Gynecology and Obstetrics, Key Laboratory of Ministry of Education on Birth Defects and Related Diseases of Women and Children, West China Second University Hospital, Sichuan University, Chengdu; d Department of Urology, Beijing Tongren Hospital, Capital Medical University, Beijing; e Department of Internal Medicine-Neurology, General Hospital of Jizhong Energy Xingtai Mining Group Co. Ltd., Xingtai; f Department of Andrology, Fucheng Technical Service Center of Family Planning, Hengshui; g Department of Andrology, Nanjing General Hospital of Nanjing Military Command, PLA, Nanjing; h Reproductive Health Research Institute, Tongji Medical College, Huazhong University of Science and Technology, Wuhan; i National Health and Family Planning Commission Key Laboratory of Male Reproductive Health, Department of Male Clinical Research, National Research Institute for Family Planning, Beijing, China.

**Keywords:** aging, hypogonadism, male, prevalence, testosterone

## Abstract

To investigate the age-related nomograms and change trends of reproductive hormones, and prevalence of androgen deficiency (AD) in middle-aged and aging men from 2 studies.

Two cross-sectional studies were conducted at 5-year intervals in Chinese community-dwelling men living in the same area. A total of 434 (Study 1, S1) and 944 (Study 2, S2) men aged 40 to 69 years were recruited as subjects and 59 (S1) and 98 (S2) men aged 20 to 39 years as controls to measure serum reproductive hormone levels.

Serum total testosterone (TT) levels did not change significantly in S1, whereas TT levels increased in S2 with aging. Serum calculated free testosterone (cFT) levels gradually decreased with aging; however, only men aged 40 to 69 years showed this trend in S2. Serum luteinizing hormone (LH) and sex hormone binding globulin (SHBG) levels gradually increased, and serum testosterone secretion index (TSI) and free testosterone index (FTI) levels gradually decreased with male aging. The mean annual decrease values of serum cFT were 2.705 pmol/l in S1 and 1.060 pmol/l in S2. The cut-off values for AD in S1 and S2 were 9.13 nmol/l and 9.35 nmol/l for TT, and 169.00 pmol/l and 213.90 pmol/l for cFT. Using TT or cFT cut-off values, mean AD prevalence was 14.52% or 44.70% in S1, and 6.36% or 16.53% in S2. Based on cFT cut-off values, prevalence of AD increased gradually with male aging in a range of 25.30% to 61.63% in S1 and 1.20% to 23.03% in S2.

The change patterns of serum LH, SHBG, TSI and FTI levels in middle-aged and aging males were consistent; however, there were differences in serum TT and cFT change patterns in S1 and S2 with male aging. cFT cut-off values were the optimal metric to evaluate AD, which can be present a ladder-like change in prevalence of different age groups.

## 1 Introduction

Change in serum reproductive hormone levels and morbidity related to late-onset hypogonadism (LOH) in middle-aged and aging males is a slow and gradual process, and the deficiency of serum testosterone with male aging was one of the main etiologies and mechanisms on LOH. According to the existing literatures, prevalence of LOH ranges from 2.1% to 40%,^[1–9]^ and it is generally agreed that serum reproductive hormone levels and LOH prevalence fluctuate with aging, however, it remains a conundrum and exists dispute on accurate prevalence and cut-off of androgen deficiency (AD) in aging males from different countries and regions.^[10–15]^ Based on the two reports from the United States and the UK, prevalence of hypogonadism is estimated to be 39% in men aged 45 years or older, and this number is likely to increase.^[16]^ Hypogonadism is more prevalent in older men and is directly related to obesity, insulin resistance, hypertension, hyperlipidemia, endothelial dysfunction in aging men, and to comorbidities and poor health status.^[13]^ Prospective data from the European Male Aging Study (EMAS) on 2599 community-dwelling men aged 40 to 79 years show that 5.7% died during a median follow-up of 4.3 years and 2.1% were identified as having LOH.^[17]^

At present, scholars have used different criteria to perform epidemiological studies on AD and LOH, and their results have produced clear differences because it is very difficult to unify the investigation methods across studies, the cut-off values used to define AD and the diagnostic criteria for LOH worldwide, and the study samples recruited from variant populations of different races, diets and cultures. The definitions of LOH, AD, testosterone deficiency and biochemical hypogonadism are not fully differentiated in the existing literature. In the same way, it has been difficult to compare the rates of prevalence or incidence across studies.

In general, AD can see as the basis of LOH morbidity. Researchers agree that AD and LOH influence health status, poorer self-rated health and frailty of middle-aged and aging males. Therefore, prevalence of AD and LOH in healthy males deserves attention. In order to investigate the age-related nomograms and change trends of reproductive hormones, and prevalence of AD in Chinese middle-aged and aging males, we performed two community-dwelling population-based cross-sectional studies in the same population at 5-year intervals. The first study (Study 1, S1) was a preliminary trial concerning an epidemiological investigation of the age-related nomograms and AD. This study investigated the prevalence of AD in Chinese men while validating research methods. The second study (Study 2, S2) was part of nationwide, multicenter trial on the reproductive health status of middle-aged and aging men. The two studies performed in same area by skilled investigators in S1 allowed for a smooth implementation of S2.

## 2 Methods

### 2.1 Study design

Two studies were cross-sectional surveys of 1560 (S1) and 1200 (S2) community-dwelling men aged 20 to 69 years and 20 to 89 years selected via cluster and age-stratified sampling in Fucheng County, Hebei Province. Each survey identified the local population register to provide a sampling frame from which participants aged 40 to 69 years were randomly selected at a scale of 10:1. The scale of 1:1 was performed to recruit the participants aged 40 to 69 years come from township and from rural area. The numbers of each age group and total numbers of each study were 250 men and 1500 men in S1, and 160 men and 960 men in S2, respectively. However, there was difference between the sample numbers of study design and the final numbers included into S1 or S2 studies when two studies conducted. Participants were recruited for each study between August 2007 and November 2008 (S1) and between July 2013 and January 2014 (S2) after written informed consent was obtained.

### 2.2 Participants

Participants were invited to complete interviewer-assisted questionnaires and to undergo a general physical examination, body measurement and blood tests for biochemical and hormone levels. The exclusion criteria were defined as follows:

1) previously or currently diagnosed malignancies, corticosteroid use, or presence of liver cirrhosis;
2) testosterone supplement or androgen-deprivation therapy use, 5-α reductase inhibitor treatment, or history of orchiectomy; and
3) current hypothalamus-pituitary disease.

In order to compare the data of the same age groups, 1498 (S1) and 944 (S2) participants aged 40 to 69 years were included in the data analysis and enrolled subjects. Fifty-nine (S1) and 98 (S2) participants aged 20 to 39 years were enrolled as controls.

### 2.3 Questionnaires

Each participant completed a questionnaire including information concerning sociodemographic and general health status, lifestyle, medical conditions, medications and two screening scales of LOH (the Questionnaire for Androgen Deficiency in Aging Males, ADAM and the Aging Males’ Symptoms Scale, AMS). Alcohol consumption was defined as one or more alcoholic drinks, including beer, wine, and spirits, per week. Smoking status was classified as never smoked or smoker.

### 2.4 Clinical and laboratory measurements

A single fasting venous blood sample was obtained from each participant in the morning (before 9 AM), and serum samples were stored in aliquots at −70°C until the time of assay. Serum samples were measured together in batches in the central laboratory of the Beijing Coordinating Center.

Serum measurements of S1: Out of 1498 participants, only 434 men were sequentially recruited as subjects and 59 men as controls to measure the concentrations of serum total testosterone (TT), luteinizing hormone (LH) (MPAIA kits from Beijing Bio-Ekon Biotechnology Co. Ltd., Beijing, China), and sex hormone binding globulin (SHBG) (ELISA kits from Diagnostic Systems Laboratories, Inc., DSL; TX), because the budget for purchasing kits was limited. The sensitivity of the TT and LH kits was 0.3 nmol/l, and 0.2 IU/l, respectively. The variable coefficients (VCs) of intra-assay were 1.8%, and 4.9%, respectively. The inter-assay VC less than 8.6%. The sensitivity of the SHBG kits was 0.61 nmol/l, and the intra- and inter-assay VCs were 6.67%, and 9.78%, respectively. cFT was calculated using equations described by Vermeulen et al.^[18]^ Furthermore, the testosterone secretion index (TSI) and free testosterone index (FTI) were calculated using the formulas TT/LH (nmol/IU) and TT/SHBG (nmol/nmol), respectively.

Serum measurements of S2: TT, SHBG, and LH concentrations in 944 subjects and 98 participants recruited as controls were measured using a Beckman UniCel DXI800 automatic chemiluminescence immune analyzer (Beckman Coulter, Fullerton, CA). The lower limits of the TT, SHBG, and LH levels were 0.35 nmol/l, 0.017 nmol/l, and 0.2 IU/l, respectively. The intra-assay VCs for TT, SHBG, and LH were 2.7%, 4.8%, and 3.8%, respectively. The mean inter-assay VCs for TT, SHBG, and LH were 5.6%, 5.3%, and 6.4%, respectively. The calculation methods for cFT, TSI and FTI were the same as used in S1.

### 2.5 Statistical analysis

The data obtained from the 2 studies were analyzed using SPSS21.0 (International Business Machines Corp., Armonk, NY). As a result of skew in the hormone distributions, we analyzed hormone data using nonparametric statistics, such that the median (50% percentile), and the 10% and 90% percentiles represented hormone mean level, the lower and higher reference limits, respectively. Hormone levels and other enumeration data from the different groups were compared using a Kruskal–Wallis H test on multiple sets of data or a Mann–Whitney *U* test on two sets of data. The correlations between hormone levels and age, serum LH and SHBG levels were analyzed using Spearman's correlation. Prevalence of AD in the different age groups, and different cut-off value were compared using a Chi-square (χ^2^) test. Results were considered statistically significant if null hypotheses could be rejected at the 0.05 level.

### 2.6 Ethics and informed consent statement

Both studies and the accompanying consent forms were approved by the Ethics Committee and Institutional Review Board of the affiliation. Participants were recruited for each study after written informed consent was obtained.

## 3 Results

### 3.1 Subject characteristics

The characteristics of the subjects are shown in Table 1. The prevalence of cardiovascular disease, other chronic diseases, on medications, alcohol rate, and smoke rate in the S1 and S2 subjects were 22.58% vs 33.79%, 39.86% vs 41.53%, 32.95% vs 35.49%, 70.05% vs 60.17%, 51.84% vs 55.19%, respectively.

**Table 1:**
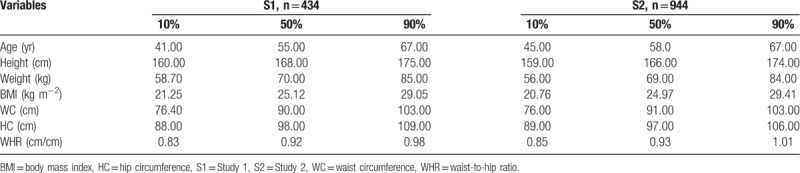
Characteristics of Study 1 subjects and Study 2 subjects.

### 3.2 Reproductive hormone nomograms and their change patterns

S1 data showed that serum TT levels did not change significantly with male aging, whereas serum LH and SHBG levels gradually increased, and cFT, TSI, FTI levels gradually decreased with male aging. Kruskal–Wallis H tests showed that there were significant differences among the seven age groups in the levels of the other 5 reproductive hormones (*P* = .000), except for serum TT levels (*P* *>* .05).

**Figure 1:**
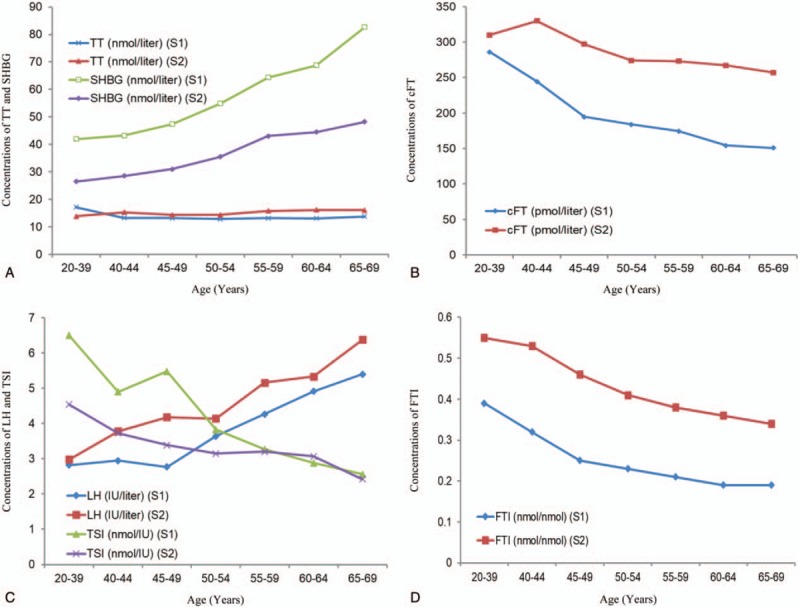
The change pattern of median reproductive hormone values with male aging. (A) The change pattern of serum TT and SHBG; (B) The change pattern of serum cFT; (C) The change pattern of serum LH and TSI; (D) The change pattern of serum FTI. cFT = calculated free testosterone, FTI = free testosterone index, LH = luteinizing hormone, SHBG = sex hormone binding globulin, TSI = testosterone secretion index, TT = total testosterone.

S2 data showed that serum LH and SHBG levels gradually increased, whereas serum TSI and FTI levels gradually decreased with male aging. Kruskal–Wallis H tests showed that there were significant differences in the levels of all 6 reproductive hormones among the seven age groups (*P* = .000). The TT median showed an increasing trend with aging, and the cFT median gradually decreased with aging in the three groups of 40 to 69 years. Unexpectedly, the cFT median of the control group fell in between the 40 to 44 years group and the 45 to 49 years group. No differences were found in cFT levels between the control group and the 40 to 44, 45 to 49 years groups (*P* = .067, *P* = .537), but there was a significant difference between the 40 to 44 and 45 to 49 years groups (*P* = .006). The change patterns of TT and cFT in S2 clearly differed from S1. The data are presented in Table 2, Table 3 and Figure 1.

**Table 2:**
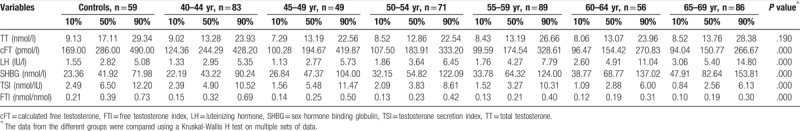
Distribution of serum reproductive hormone levels of Study 1 in different age groups (10%, 50%, and 90% percentiles).

**Table 3:**
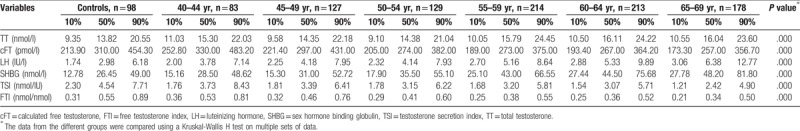
Distribution of serum reproductive hormone levels of Study 2 in different age groups (10%, 50% and 90% percentiles).

### 3.3 The change velocity of medium serum hormone values with male aging

Mean change velocity was calculated using the formula: [(median of 65–69 years group – median of 40–44 years group)/30 years]. Data from S1 and S2 indicated that mean annual decrease values of serum cFT were 2.705 and 1.060 pmol/l per year, respectively; and cFT levels progressed into a variation acceleration phase in the 45 to 49 years group.

### 3.4 Analysis of correlations between age, LH, SHBG, and reproductive hormones

Spearman's correlation indicated that, in both S1 and S2, there were positive correlations between age and serum LH, SHBG, and between serum LH and SHBG, and serum SHBG and TT (*P* *<* .001); moreover, there were negative correlations between age, serum LH and cFT, TSI, FTI, and between serum SHBG and cFT, FTI (*P* *<* .001). There were positive correlations between age, serum LH and TT in S2 (*P* *<* .001).

### 3.5 Serum hormone cut-off values for AD diagnosis

AD was defined as a serum testosterone concentration below the cut-off value, irrespective of subjects with or without LOH symptoms in our 2 studies. Using the 10% percentile of serum hormone levels in controls (20–39 years group) as the cut-off value, cut-off values for AD in S1 and S2 were 9.13 nmol/l and 9.35 nmol/l for serum TT, 169.00 pmol/l and 213.90 pmol/l for serum cFT, respectively.

### 3.6 Prevalence of AD in subjects and comparison between different age groups

The characteristics of AD prevalence are presented in Table 4. When the serum cFT cut-off value was used, AD prevalence increased gradually with male aging and ranged from 25.30% to 61.63% in S1 and from 1.20% to 23.03% in S2. However, if the serum TT cut-off value was used, AD prevalence did not show an increasing trend with aging. χ^2^ tests indicated that there were significant differences in prevalence of AD among the 6 age groups using the cFT cut-off value (*P* *<* .01).

**Table 4:**
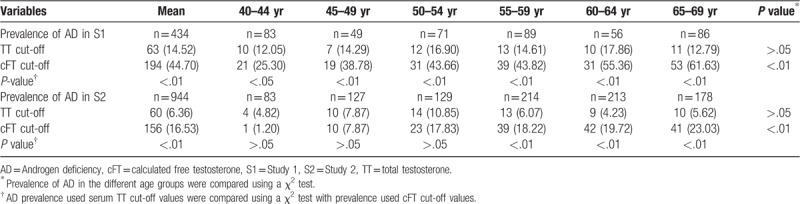
Prevalence of Androgen deficiency in different age groups, n (%).

Compared AD prevalence used serum TT cut-off values with prevalence used cFT cut-off values, there were significant differences in each age group of S1 (40–44 years group: *P* *<* .05; the other 5 age groups and mean prevalence: *P* *<* .01), and there were significant differences in the 55–59, 60–64, and 65–69 years groups of S2 (*P* *<* .01).

## 4 Discussion

In our 2 cross-sectional studies, the change patterns of serum LH, SHBG, TSI and FTI levels in middle-aged and aging males were consistent, unexpectedly, the change patterns of serum TT and cFT levels were not consistent in S1 and S2. We found that instead of decreasing, serum TT level showed an increasing tendency with aging in S2; serum cFT levels showed a decreasing tendency with aging, however, only subjects aged 40 to 69 years had this tendency in S2. The reason for the inconsistency in serum TT and cFT production may be attributed to sampling bias and confounding factors. A previous study reported that the mean serum TT level did not decline significantly with aging during a median 4.3-year follow-up (18.1 vs 17.8 nmol/l) and that the longitudinal change in serum TT was approximately +0.8% per year,^[19]^ which was similar to our S2 findings. However, other studies agreed that significant differences in serum TT, LH, SHBG, FTI, and TSI levels were found between different age groups and that serum TT levels in middle-aged and aging men were significantly decreased.^[1,20–23]^ Interestingly, 2 reports were consistent with our S1 results, showing that there were no age-related changes in serum TT in healthy men, but serum cFT levels did exhibit age-related changes.^[24,25]^ The dynamic alteration of serum TT with aging remains controversial, but similar controversies exist regarding the alteration patterns of other hormones.

Our results indicated that serum cFT progressed into a variation acceleration phase in the 45 to 49 years group. With a rapid decrease in serum cFT, the prevalence of AD rapidly increased in the same group in S1 (from 25.30% in the 40–45 years to 38.78% in the 45–49 years) and in the 50 to 54 years group in S2 (from 7.87% in the 45–49 years to 17.83% in the 50–54 years), a finding that has been reported by other researchers.^[25]^ Several studies reported one measure of velocity expressed as an annual variation value. Two reports reported annual changes as follows: −0.1 ± 0.95 nmol/l for TT, −3.83 ± 16.8 pmol/l for free testosterone (FT);^[22]^ −0.124 nmol/l for TT.^[1]^ The second kind of velocity measure was expressed as an annual variation percentage; for example, the estimated cross-sectional decline in TT levels was −0.4% per year of age, the longitudinal within-subject decline was −1.6% per year, and the age-matched time trend was −1.2% per year.^[20]^ Another longitudinal analysis showed that the TT of men older than 60 years declined by approximately 1.3% and 0.9% per annum.^[21]^ The annual velocities of cFT in the S1 and S2 results were less than in the existing literature (−3.12 pmol/l for FT).^[11]^

In light of the significant differences in the levels and change patterns of serum TT, cFT, LH, SHBG, TSI and FTI in S1 and S2, we suggest that the differences may be due to the following reasons:

1) subjects of S1 and S2 were recruited over a 5-year interval in the same population;
2) sample sizes differed between S1 and S2;
3) measurement methods in serum TT, LH and SHBG differed;
4) there were differences in subjects’ ages; and
5) differences existed with respect to weight, WHR, alcohol consumption and cardiovascular status.

The European Association of Urology (EAU) guidelines on LOH set forth the following recommendations: both immunoassay- and mass spectrometry-based assays can produce reliable TT results as long as they are well validated; evaluation of LOH and AD should be based on reference ranges for normal men provided by the laboratory measuring the samples; and the calculation of FT based on serum SHBG concentration is recommended for determination of serum FT levels.^[26]^ In our studies, different categories of kits were used to measure serum hormones and SHBG concentrations, therefore, measurement inconsistencies were inevitable across the two studies.

The data suggest decreased serum testosterone levels could be one of manifestations comorbid factors influenced. The prevalence of AD and LOH may be reduced by prevention or treatment of comorbidity. Reduced serum TT and estimated free testosterone in hypogonadal men were observed in 34% and 47% of systolic heart failure (HF) patients, respectively, and 15% of men with HF were diagnosed with LOH.^[27]^ The differences in comorbidities and body measurement parameters from our results may influence hormone levels and AD prevalence of the subjects.

Although the prevalence of LOH closely correlates with the prevalence of AD, the relationship is not symmetrical because only some men with AD manifest symptoms of LOH, and only symptomatic AD patients are diagnosed with LOH. It was clear that the cut-off values of serum TT and cFT in Chinese men, especially in S1 subjects, are notably lower than those of the EMAS study (11 nmol/l for TT, 220 pmol/l for FT) and the EAU guidelines (12.1 nmol/l for TT, 243 pmol/l for FT),^[7,26]^ and the thresholds for detecting men with AD-related symptoms (10.4 nmol/l for TT, 225 pmol/l for cFT).^[28]^ Comparing these data with our results, only the cut-off value of serum cFT in S2 is close to the EMAS and EAU values. In addition, we found that the serum FT cut-off value was close to optimal for screening, evaluating and diagnosing AD, and was suitable for different age groups due to the ladder-like change patterns of serum FT. AD prevalence increased gradually with male aging, which showed a real and potential prevalence trend when using the serum cFT cut-off value. Although EAU guidelines suggest using serum TT to diagnose AD and reserving use of serum cFT only for men with borderline serum TT, Antonio et al^[29]^ robustly demonstrated that low serum cFT, even in the presence of normal serum TT, was associated with AD-related symptoms; however, normal serum cFT, despite low serum TT, was not associated with cognate symptoms. In brief, it should be more precise and reliable to use serum FT levels to evaluate the AD and LOH status of aging males; the serum FT cut-off value is more valuable and more significant than the TT cut-off value.

## 5 Conclusions

Analyzed the data from our 2 studies, we found the change patterns of serum LH, SHBG, TSI and FTI levels in middle-aged and aging males were consistent, serum LH and SHBG levels gradually increased, and serum TSI and FTI levels gradually decreased with male aging. However, serum TT levels did not change significantly in S1, and TT levels increased in S2 with aging. Serum cFT levels gradually decreased with aging; however, only men aged 40 to 69 years showed this trend in S2. Using the serum cFT cut-off value, mean AD prevalence of S1 and S2 was 44.70% and 16.53%, respectively, which was higher than some reports in the literatures. Serum cFT cut-off values were near optimal for to evaluating AD, which could present the ladder-like change pattern of AD prevalence in different age groups.

### 5.1 Limitations

In comparison with American and European studies, the two studies presented here still pose some shortcomings. For example, the laboratory measurements of serum TT, SHBG, and LH used different methods in the two studies, which was one of several reasons that lead to significant differences in serum TT, cFT, LH, and SHBG levels between S1 and S2. Furthermore, the differences of hormone levels could influence the AD cut-off values and thus the prevalence of AD. In addition, there was an inconsistency in the sample size and the significant differences of age, WHR, lifestyle and medical conditions between participants in the two groups. These limitations may affect research findings, which will be taken into consideration in designing and conducting further studies. Although our two studies have some limitations, valuable knowledge can still be gleaned by our research team and future researchers.

## Acknowledgments

The authors wish to thank all participants and their families for participating in this study. We gratefully acknowledge the help of Dr. Ru-Ming Shu, Can-Gang Wang, Li-Hua Zhuang and other staff of Fucheng Technical Service Center of Family Planning for their excellent technical assistance in participants recruitment, questionnaire management, and the collection, storage and transport of blood samples. And we gratefully acknowledge the help of Dr Dian He for the statistical analysis, Dr He was from Department of Epidemiology and Health Statistics, School of Public Health, Capital Medical University, Beijing, China.

## Author contributions

**Conceptualization:** Shan-Jie Zhou, Yi-Qun Gu.

**Data curation:** Shan-Jie Zhou, Yi-Qun Gu.

**Formal analysis:** Shan-Jie Zhou, Yi-Qun Gu.

**Funding acquisition:** Shan-Jie Zhou, Xue-Jun Shang, Cheng-Liang Xiong, Yi-Qun Gu.

**Investigation:** Shan-Jie Zhou, Ming-Jia Zhao, Yi-Hong Yang, Di Guan, Zhi-Guang Li, Yu-Dang Ji, Bao-Long Zhang.

**Methodology:** Shan-Jie Zhou, Xue-Jun Shang, Cheng-Liang Xiong, Yi-Qun Gu.

**Project administration:** Shan-Jie Zhou, Xue-Jun Shang, Cheng-Liang Xiong, Yi-Qun Gu.

**Resources:** Xue-Jun Shang, Cheng-Liang Xiong, Yi-Qun Gu.

**Supervision:** Xue-Jun Shang, Cheng-Liang Xiong, Yi-Qun Gu.

**Validation:** Xue-Jun Shang, Cheng-Liang Xiong, Yi-Qun Gu.

**Visualization:** Xue-Jun Shang, Cheng-Liang Xiong, Yi-Qun Gu.

**Writing – original draft:** Shan-Jie Zhou, Yi-Qun Gu.

**Writing – review & editing:** Shan-Jie Zhou, Yi-Qun Gu.
